# 贝林妥欧单抗桥接CAR-T细胞疗法治疗成人急性B淋巴细胞白血病疗效及安全性分析

**DOI:** 10.3760/cma.j.cn121090-20231127-00283

**Published:** 2024-04

**Authors:** 妍 浦, 湘粤 周, 吟 刘, 欣 孔, 晶晶 韩, 剑 张, 志洪 林, 君 陈, 惠英 仇, 德沛 吴

**Affiliations:** 1 苏州大学附属第一医院血液内科，江苏省血液研究所，国家血液系统疾病临床医学研究中心，苏州 215006 The First Affiliated Hospital of Soochow University, Jiangsu Institute of Hematology, National Clinical Research Center for Hematologic Diseases, Suzhou 215006, China; 2 苏州永鼎医院血液科，苏州 215200 Hygeia Suzhou Yongding Hospital, Suzhou 215200, China

**Keywords:** 贝林妥欧单抗, 嵌合抗原受体T细胞, 急性B淋巴细胞白血病, 抗肿瘤免疫治疗方案, 微小残留病, Blinatumomab, Chimeric antigen receptor T-cell, Leukemia, B-cell lymphoblastic, acute, Anti-tumor Immunotherapy, Minimal residual disease

## Abstract

**目的:**

探讨贝林妥欧单抗桥接嵌合抗原受体T（CAR-T）细胞疗法治疗急性B淋巴细胞白血病（B-ALL）患者的疗效及安全性。

**方法:**

回顾性分析2018年8月至2023年5月在苏州大学附属第一医院住院治疗的36例成人B-ALL患者的临床资料。男18例，女18例，中位年龄为43.5（21～72）岁。其中费城染色体阳性急性淋巴细胞白血病（Ph^+^ALL）21例，复发/难治16例。18例患者接受贝林妥欧单抗桥接CAR-T细胞治疗，18例患者仅接受CAR-T细胞治疗，分析两组患者的疗效及安全性。

**结果:**

贝林妥欧单抗桥接CAR-T组中16例患者贝林妥欧单抗治疗后获得完全缓解（CR），CR率88.9％。桥接CAR-T治疗1个月后复查骨髓象，CR率为100.0％，微小残留病（MRD）阴性率高于未接受贝林妥欧单抗桥接治疗组（94.4％对61.1％，Fisher，*P*＝0.041）。贝林妥欧单抗桥接CAR-T组患者细胞因子释放综合征（CRS）及其他不良反应发生率低于未接受贝林妥欧单抗桥接治疗组（11.1％对50.0％，Fisher，*P*＝0.027）。截至随访终点，贝林妥欧单抗桥接CAR-T组中13例患者持续MRD阴性状态，5例患者（包含2例经贝林妥欧单抗治疗无效的病例）在治疗后2.57～10.20个月复发，2例复发患者后续接受第二次CAR-T细胞治疗后达CR。未接受贝林妥欧单抗桥接治疗组中10例患者持续MRD阴性状态，7例复发，6例死亡。贝林妥欧单抗桥接CAR-T组患者1年总生存率高于未接受桥接治疗组，差异在0.1水平有统计学意义［（88.9±10.5）％对（66.7±10.9）％，*P*＝0.091］。

**结论:**

贝林妥欧单抗桥接CAR-T细胞治疗成人B-ALL疗效及安全性值得肯定，清除肿瘤残留效果好，近期疗效复发率低，不良反应少。

## Abstract (en)

**Objective:**

Exploring the efficacy and safety of bridging blinatumomab（BiTE）in combination with chimeric antigen receptor T（CAR-T）cell therapy for the treatment of adult patients with acute B-cell lymphoblastic leukemia（B-ALL）.

**Methods:**

Clinical data from 36 adult B-ALL patients treated at the First Affiliated Hospital of Suzhou University from August 2018 to May 2023 were retrospectively analyzed. A total of 36 cases were included: 18 men and 18 women. The median age was 43.5 years (21–72 years). Moreover, 21 cases of Philadelphia chromosome-positive acute lymphoblastic leukemia were reported, and 16 of these cases were relapsed or refractory. Eighteen patients underwent blinatumomab bridging followed by CAR-T cell therapy, and 18 patients received CAR-T cell therapy. This study analyzed the efficacy and safety of treatment in two groups of patients.

**Results:**

In the BiTE bridge-to-CAR-T group, 16 patients achieved complete remission（CR）after BiTE immunotherapy, with a CR rate of 88.9％. One month after bridging CAR-T therapy, bone marrow examination showed a CR rate of 100.0％, and the minimal residual disease（MRD）negativity rate was higher than the nonbridging therapy group（94.4％ *vs*. 61.1％, Fisher, *P*＝0.041）. The incidence of cytokine release syndrome and other adverse reactions in the BiTE bridge-to-CAR-T group was lower than that in the nonbridging therapy group（11.1％ *vs*. 50.0％, Fisher, *P*＝0.027）. The follow-up reveals that 13 patients continued to maintain MRD negativity, and five patients experienced relapse 8.40 months（2.57–10.20 months）after treatment. Two of five patients with relapse achieved CR after receiving the second CAR-T cell therapy. In the nonbridging therapy group, 10 patients maintained continuous MRD negativity, 7 experienced relapse, and 6 died. The 1 year overall survival rate in the BiTE bridge-to-CAR-T group was higher than that in the nonbridging therapy group, with a statistically significant difference at the 0.1 level［（88.9±10.5）％ *vs*.（66.7±10.9）％, *P*＝0.091］.

**Conclusion:**

BiTE bridging CAR-T cell therapy demonstrates excellent efficacy in adult B-ALL treatment, with a low recent recurrence rate and ongoing assessment of long-term efficacy during follow-up.

目前，成人急性B淋巴细胞白血病（B-ALL）的治疗仍然是血液肿瘤领域中的一个重大挑战。嵌合抗原受体T（CAR-T）细胞免疫疗法的出现改善了复发/难治性急性B淋巴细胞白血病（r/r B-ALL）患者的预后。贝林妥欧单抗是一种双特异性抗CD3和抗CD19单克隆抗体，获批用于治疗r/r B-ALL和微小残留病灶（MRD）阳性的B-ALL，可实现更深的缓解并改善患者生存。有研究表明，贝林妥欧单抗治疗r/r B-ALL的疗效优于常规化疗，然而贝林妥欧单抗的作用不持久，桥接造血干细胞移植（HSCT）治疗后仍有部分患者复发[1]。本研究我们应用贝林妥欧单抗桥接CAR-T细胞联合免疫治疗18例B-ALL患者，5例患者进一步实施HSCT，现将疗效及安全性报道如下。

## 病例与方法

1. 一般资料：回顾性分析2018年8月至2023年5月苏州大学附属第一医院36例接受CAR-T细胞治疗的成人B-ALL患者的临床资料，诊断符合2022版WHO造血和淋巴组织肿瘤的分型诊断标准[2]。所有患者经骨髓细胞形态学、白血病免疫分型、细胞遗传学、分子生物学等检查确诊。本研究定义高危因素包括年龄>35岁、初诊WBC>30×10^9^/L、高危融合基因阳性或基因突变（BCR::ABL、ABL类、TP53、JAK STAT、PAX5、IKZF1）、高危染色体异常（复杂核型、21号染色体内部扩增）、髓外浸润。

2. 治疗方案：36例B-ALL患者中，18例接受贝林妥欧单抗桥接CAR-T细胞治疗，18例患者CAR-T细胞治疗前未予桥接治疗。贝林妥欧单抗治疗方案：9 µg/d、d 1～2，28 µg/d、d 3～14（17例）或9 µg/d、d 1～7，28 µg/d、d 8～28（1例）。16例患者贝林妥欧单抗治疗有效，疗程结束后第2天输注CAR-T细胞；2例患者贝林妥欧单抗免疫治疗后未缓解，原幼细胞比例分别为20.0％、57.5％，疗程结束后间隔1周行FC方案（氟达拉滨30 mg·m^−2^·d^−1^、−6～−3 d，环磷酰胺300 mg·m^−2^·d^−1^、−6～−3 d）预处理清除淋巴细胞后输注CAR-T细胞。CAR-T细胞输注量为1×10^7^/kg，靶标CD19/CD22，分3次输注，第1天10％、第2天30％、第3天60％。18例未接受贝林妥欧单抗桥接治疗的B-ALL患者FC方案预处理后输注CAR-T细胞，CAR-T细胞的中位输注量为1（0.5～1）×10^7^/kg，5例靶标为CD19/CD22，13例靶标为CD19，分3次输注，第1天10％、第2天30％、第3天60％。费城染色体阳性急性淋巴细胞白血病（Ph^+^ALL）患者治疗后予以酪氨酸激酶抑制剂（TKI）维持治疗。CAR-T细胞均由上海优卡迪生物医药科技有限公司制备，共刺激域为4-1BB、CD19和CD22的单链抗体均为鼠源，CD19 CAR-T同时携带IL6 shRNA敲减元件。

3. 疗效评估：分别在贝林妥欧单抗免疫治疗前、治疗后及CAR-T细胞治疗后复查骨髓，应用多参数流式细胞术（FCM）检测患者的骨髓MRD水平。对于融合基因阳性的患者，同时应用实时定量PCR（RT-PCR）检测MRD。对于费城染色体阴性的患者，MRD阳性定义为FCM结果≥1×10^−4^，对于费城染色体阳性的患者，MRD阳性定义为RT-PCR结果BCR::ABL1/ABL1≥0.01％。B-ALL难治、复发的定义参照《中国成人急性淋巴细胞白血病诊断与治疗指南（2021年版）》[3]。动态监测CAR-T拷贝数评估患者CAR-T细胞扩增水平。

4. 不良反应评估：不良反应中血液学毒性及非血液学毒性依据CTCAE 5.0标准[4]，细胞因子释放综合征（CRS）分级依据Lee分级标准[5]。

5. 随访：采用电话、查阅患者住院及门诊病历的方式随访，随访截止日期为2023年11月24日，中位随访8.13（1.67～16.13）个月，所有患者均未失访。总生存（OS）期定义为自贝林妥欧单抗或FC预处理方案至患者死亡或随访终点的时间。

6. 统计学处理：采用R 4.2.3软件进行统计学分析。分类变量以例数（百分比）描述，采用Fisher精确概率法进行组间比较；连续变量以中位数（范围）描述，组间比较采用秩和检验（不符合正态分布）；应用Kaplan-Meier方法进行生存分析，双侧*P*<0.05为差异有统计学意义。

## 结果

1. 一般资料：共纳入36例成人B-ALL患者，其中男18例，女18例，中位年龄43.5（21～72）岁。初诊中位WBC 15.6（0.8～416.0）×10^9^/L。21例患者为Ph^+^ ALL，其中5例为慢性髓性白血病（CML）（急淋变）。治疗前患者中位骨髓原始细胞比例为1.75％（0～74.50％）。8例患者存在高危基因突变，其中2例伴TP53突变，1例同时伴TP53突变和IKZF1改变，1例伴ABL类突变，2例伴JAK STAT突变，2例伴PAX5突变。5例伴BCR::ABL融合基因且既往CML。5例患者为复杂核型。16例（44.4％）患者为复发/难治性ALL。既往接受的中位治疗线数为3（1～7）线，3例（8.3％）患者接受过HSCT。贝林妥欧单抗桥接CAR-T组及未接受贝林妥欧单抗桥接治疗组患者主要临床特征见表1，后者高危基因突变的比例更高（5.6％对38.9％，*P*＝0.041），其他临床特征差异均无统计学意义（均*P*>0.05）。

**表1:** 36例接受嵌合抗原受体T细胞（CAR-T细胞）治疗B-ALL患者基本临床特征

患者特征	BiTE+CAR-T组（18例）	FC+CAR-T组（18例）	统计量	*P*值
性别[例（%）]			0.111	0.739
男	8（44.4）	10（55.6）		
女	10（55.6）	8（44.4）		
是否Ph阳性[例（%）]			1.829	0.176
否	10（55.6）	5（27.8）		
是	8（44.4）	13（72.2）		
年龄[例（%）]			Fisher	0.177
≤35岁	5（27.8）	1（5.6）		
>35岁	13（72.2）	17（94.4）		
初诊WBC[例（%）]			0.113	0.737
≤30×10^9^/L	9（50.0）	11（61.1）		
>30×10^9^/L	9（50.0）	7（38.9）		
治疗前疾病状态[例（%）]			1.013	0.314
复发/难治	6（33.3）	10（55.6）		
非复发/难治	12（66.7）	8（44.4）		
高危因素[例（%）]	3（16.7）	10（55.6）	Fisher	0.035
高危基因突变	1（5.6）	7（38.9）	Fisher	0.041
高危染色体异常	2（11.1）	3（16.7）	Fisher	1.000
既往CML	3（16.7）	2（11.1）	Fisher	1.000

**注** BiTE：贝林妥欧单抗；FC：预处理方案，氟达拉滨30 mg·m^−2^·d^−1^、−6～−3 d，环磷酰胺300 mg·m^−2^·d^−1^、−6～−3 d；Ph：费城染色体；CML：慢性髓性白血病

2. 疗效评价：36例B-ALL患者在疾病的不同阶段接受CAR-T细胞治疗，分别于治疗前及治疗后1个月复查骨髓象，其中贝林妥欧单抗桥接CAR-T组CR率为100.0％（18/18），MRD阴性率为94.4％（17/18），5例患者后续接受HSCT；未接受贝林妥欧单抗桥接治疗组CR率为94.4％（17/18），MRD阴性率为61.1％（11/18），2例患者后续接受HSCT。贝林妥欧单抗桥接CAR-T组患者接受CAR-T细胞治疗后MRD阴性率高于未接受贝林妥欧单抗桥接治疗组（Fisher，*P*＝0.041）。贝林妥欧单抗桥接CAR-T组中，16例患者接受贝林妥欧单抗治疗后达CR（13例达MRD阴性），2例未缓解患者CAR-T细胞治疗后均达CR，1例达MRD阴性。13例患者持续MRD阴性状态，5例（包含2例经贝林妥欧单抗治疗无效的病例）在治疗后8.40（2.57～10.20）个月复发。5例复发患者中2例死亡，2例后续接受二次CAR-T细胞治疗后达CR，1例带病生存。未接受贝林妥欧单抗桥接治疗组中10例患者持续MRD阴性状态，7例复发，6例死亡。贝林妥欧单抗桥接CAR-T组患者1年OS率高于未接受桥接治疗组，差异在0.1水平有统计学意义［（88.9±10.5）％对（66.7±10.9）％，*P*＝0.091］。动态监测患者的CAR-T拷贝数（表2），18例患者均有不同程度的扩增，中位达峰时间为9（3～20）d。

**表2:** 18例急性B淋巴细胞白血病患者接受贝林妥欧单抗（BiTE）桥接CAR-T细胞治疗的疗效及CAR-T扩增情况

例号	BiTE前		BiTE后CAR-T前		CAR-T后		CAR-T拷贝数峰值	CAR-T扩增倍数	达峰时间（d）
	骨髓形态	MRD	骨髓形态	MRD	骨髓形态	MRD			
1	CR	阴性	CR	阴性	CR	阴性	1.23×10^2^	2	4
2	CR	阴性	CR	阴性	CR	阴性	9.64×10^4^	3 935	12
3	CR	阳性	CR	阳性	CR	阴性	2.06×10^5^	1 776	9
4	CR	阳性	CR	阴性	CR	阴性	2.50×10^3^	18	9
5	CR	阴性	CR	阴性	CR	阴性	3.73×10^2^	6	4
6	CR	阴性	CR	阳性	CR	阴性	5.63×10^2^	6	3
7	CR	阳性	CR	阴性	CR	阴性	9.37×10^3^	60	12
8	CR	阴性	CR	阴性	CR	阴性	1.85×10^3^	20	9
9	CR	阳性	CR	阴性	CR	阴性	6.18×10^4^	252	12
10	CR	阴性	CR	阴性	CR	阴性	6.17×10^4^	1 350	12
11	CR	阳性	CR	阴性	CR	阴性	1.53×10^3^	28	8
12	CR	阴性	CR	阴性	CR	阴性	2.23×10^4^	98	9
13	NR	NR	NR	NR	CR	阳性	1.41×10^5^	3 059	6
14	NR	NR	NR	NR	CR	阴性	3.32×10^3^	7	8
15	CR	阳性	CR	阴性	CR	阴性	7.12×10^3^	211	20
16	CR	阳性	CR	阴性	CR	阴性	6.30×10^2^	92	12
17	PR	PR	CR	阴性	CR	阴性	7.25×10^3^	11	6
18	NR	NR	CR	阳性	CR	阴性	5.41×10^2^	2	4

**注** CAR-T：嵌合抗原受体T；CR：完全缓解；PR：部分缓解；NR:：未缓解

3. 不良反应：36例B-ALL患者在应用CAR-T细胞治疗后均出现了不同程度的不良反应（表3），贝林妥欧单抗桥接CAR-T组CRS发生率低于未接受贝林妥欧单抗桥接治疗组，差异有统计学意义（11.1％对50.0％，Fisher，*P*＝0.027）。18例接受贝林妥欧桥接CAR-T细胞治疗的B-ALL患者中2例CAR-T细胞输注后出现CRS表现，其中1例为1级CRS反应，合并电解质紊乱；1例为2级CRS反应，合并肺部感染、癫痫；无3级及3级以上CRS反应发生；无CAR相关脑病综合征（CRES）发生；1例出现Ⅲ～Ⅳ级血小板、中性粒细胞减少。18例未接受贝林妥欧单抗桥接治疗的患者中，9例CAR-T细胞输注后出现CRS表现，其中1级CRS 4例，2级CRS 2例，3级CRS 3例；1例为CRES；3例评估为长期血液学毒性，2例合并有CAR-T细胞治疗相关凝血病（CARAC）；6例患者存在肺部感染或血流感染，其中3例伴电解质紊乱，2例出现呼吸衰竭、肾功能不全、感染性休克。

**表3:** 36例B-ALL患者接受CAR-T细胞治疗的不良反应

不良反应[例（%）]	BiTE+CAR-T（18例）	FC+CAR-T（18例）	*P*值
CRS	2（11.1）	9（50.0）	0.027
1~2级	2（11.1）	6（33.3）	0.229
≥3级	0	3（16.7）	0.229
CRES	0	1（5.6）	1.000
CARAC	0	2（11.1）	0.486
长期血液毒性	1（5.6）	3（16.7）	0.603
感染	1（5.6）	6（33.3）	0.088
电解质紊乱	1（5.6）	3（16.7）	0.603
器官衰竭	0	2（11.1）	0.486

**注** B-ALL：急性B淋巴细胞白血病；CAR-T：嵌合抗原受体T；CRS：细胞因子释放综合征；CRES：CAR相关脑病综合征；CARAC：CAR-T细胞治疗相关凝血病

## 讨论

贝林妥欧单抗是一种靶向CD3和CD19的双特异性T细胞抑制剂，通过促使CD3^+^ T细胞与CD19^+^ B细胞结合，激活内源性T细胞毒性潜能，使CD19阳性癌细胞的定向裂解[6]。Yoon等[1]报道显示，贝林妥欧单抗治疗r/r B-ALL患者的疗效显著优于标准化疗，可有效降低肿瘤负荷，清除MRD。Mark等[7]提出将贝林妥欧单抗免疫治疗引入巩固治疗可以改善诱导化疗后达MRD阴性的B-ALL患者的OS。贝林妥欧单抗免疫治疗在r/r B-ALL和诱导后达MRD阴性的患者中均表现出了卓越的治疗效果。然而，根据最近r/r B-ALL的长期生存数据，尽管贝林妥欧单抗治疗后的3年OS期优于传统治疗组，但仍有70％的患者复发和死亡[1]。目前，靶向CD19的CAR-T细胞治疗已经成为治疗B-ALL的有效选择[8]–[9]。两种免疫疗法联合治疗能否提高B-ALL患者的完全缓解（CR）率及MRD转阴率以及延长持续缓解的时间值得探讨。

Pillai等[10]的研究显示，贝林妥欧单抗可能导致CD19表达下调，在接受贝林妥欧单抗治疗后CD19表达阴性的患者在后续接受CAR-T细胞治疗时疗效不佳，复发风险高。Marschollek等[11]分析了7例儿童B-ALL患者接受贝林妥欧单抗免疫治疗联合CAR-T细胞治疗的临床数据，6例患者在CAR-T前达到MRD阴性；4例持续缓解且MRD阴性；3例因CD19阴性复发，2例死亡，1例经HSCT存活。目前两种免疫疗法联合治疗B-ALL报道较少。本研究接受贝林妥欧单抗桥接CAR-T细胞治疗的18例B-ALL患者中16例对贝林妥欧单抗治疗有反应，有效率高达88.9％，2例患者无反应，此2例在CAR-T细胞治疗后均达CR，提示贝林妥欧单抗和CAR-T针对CD19抗原靶标存在着不同的免疫作用，2例贝林妥欧单抗无反应的患者分别在治疗后8.4、9.2个月复发。MRD阴性率为94.4％，与未接受贝林妥欧单抗桥接治疗组相比，贝林妥欧单抗桥接CAR-T组患者MRD阳性率更高。目前仍有13例患者持续缓解和MRD阴性状态，所有18例患者的中位OS期均未达到。贝林妥欧单抗桥接CAR-T组患者1年OS率高于未接受桥接治疗组。动态监测患者的CAR-T拷贝数变化趋势，所有患者CAR-T细胞均有不同程度扩增，证实了CAR-T扩增与疾病缓解的协同作用[12]。氟达拉滨联合环磷酰胺是最广泛使用的预处理方案，氟达拉滨对分裂期和静止期的淋巴细胞都有选择性抑制作用，环磷酰胺抗瘤谱广。贝林妥欧单抗分子量小、组织渗透力强、特异性识别能力强，能够最大程度与效应T细胞结合并激活效应性T细胞。本研究中有16例患者未接受FC方案清淋治疗，也同样获得CAR-T体内扩增，说明FC方案广泛清除淋巴细胞而非特异性识别并杀伤效应T细胞，与贝林妥欧单抗免疫治疗之间可能存在协同作用。

安全性问题是CAR-T细胞治疗首要解决的问题，最常见的不良反应是CRS，CRS的严重程度与输注前的肿瘤负荷正相关，在CAR-T细胞输注前应根据患者的情况选择个体化的预处理方案以降低肿瘤负荷[13]。Nguyen等[14]的研究表明，120例ALL患者中任何级别CRS的发生率为86％，严重CRS发生率为7％，神经毒性事件发生率为12％。本研究中，18例在贝林妥欧单抗免疫治疗后接受CAR-T细胞的回输的B-ALL患者CRS的发生率仅为11.1％（2/18），低于未接受桥接治疗组，差异有统计学意义。未见严重CRS及神经毒性事件发生。在其他并发症方面，接受贝林妥欧单抗桥接CAR-T细胞治疗患者的感染、发热、电解质紊乱、凝血功能异常等不良反应的发生率均显著降低，程度轻，且无治疗相关死亡。

综上所述，本研究B-ALL患者接受贝林妥欧单抗桥接CAR-T细胞治疗的近期CR率和MRD转阴率高，持续缓解状态时间长，复发及CRS发生率低，程度轻，获得了显著的初步疗效及安全性。但本研究样本量相对较小，随访时间较短，远期疗效及安全性仍需更大样本的前瞻性研究以及更长时间的随访得以证实。
